# The limits of testing particle-mediated oxidative stress in vitro in predicting diverse pathologies; relevance for testing of nanoparticles

**DOI:** 10.1186/1743-8977-6-13

**Published:** 2009-04-27

**Authors:** Ken Donaldson, Paul JA Borm, Vincent Castranova, Mary Gulumian

**Affiliations:** 1 MRC/University of Edinburgh Centre for Inflammation Research, ELEGI Colt Laboratory, Queen's Medical Research Institute, 47 Little France Crescent, Edinburgh, EH16 4TJ, UK; 2 Zuyd University, Nieuw Eyckholt 300, Heerlen, Limburg, 6400 AN, The Netherlands; 3 National Institute for Occupational Safety and Health, 1095 Willowdale Road, Morgantown, West Virginia, USA; 4 National Institute for Occupational Health, P O Box 4788, Johannesburg 2000, University of the Witwatersrand, Witwatersrand, South Africa

## Abstract

*In vitro *studies with particles are a major staple of particle toxicology, generally used to investigate mechanisms and better understand the molecular events underlying cellular effects. However, there is ethical and financial pressure in nanotoxicology, the new sub-specialty of particle toxicology, to avoid using animals. Therefore an increasing amount of studies are being published using in vitro approaches and such studies require careful interpretation. We point out here that 3 different conventional pathogenic particle types, PM_10_, asbestos and quartz, which cause diverse pathological effects, have been reported to cause very similar oxidative stress effects in cells in culture. We discuss the likely explanation and implications of this apparent paradox, and its relevance for testing in nanotoxicology.

## The nanoparticle testing problem

It is well-recognised that nanoparticles pose a problem for toxicological testing. Nanoparticles represent a broad class of materials that have come under ever greater scrutiny, yet there is little generic evidence on which endpoints to choose to predict potential pathogenicity. Nanoparticles may cause effects like 'conventional particles' but to a greater degree because of their greater surface area per unit mass. In addition, they may translocate to the blood or brain and cause other effects. Nanoparticles come in an array of compositions, sizes, shapes and with modified surfaces, all of which could alter toxicity. The sheer number of variants that need testing has stimulated a move towards the idea that *in vitro *testing might provide an answer to the otherwise large scale animal testing, that is both expensive and time consuming. Indeed, Nel et al. [1] have proposed that generation of reactive species and induction of oxidant stress may form the basis for development of in vitro screening methodology. In contrast, Oberdorster et al. [2] summarized the views of a panel of nano scientists which placed in vivo studies as primary to the evaluation of potential health hazard of nanoparticles. We point out here the potential dangers of exclusive use of an in vitro screening approach in view of experience with other particles, suggesting that particles can cause a rather narrow range of effects on cells in culture which does not reflect the range of different pathogenic effects they cause *in vivo*. This is likely in large part due to the issue of translocation and toxicokinetics, which is one of the most under-researched areas in nanoparticle toxicology, as well as for conventional particle toxicology.

## Diverse effects of different particles in causing disease

It is an apparent paradox that exposure to different kinds of particles causes a range of different adverse effects whilst, *in vitro *at the cellular/molecular level, they have been found to influence similar pathways and mechanisms, mostly based around oxidative stress. The particles under consideration here are asbestos, quartz and PM_10 _and the different diseases they are associated with are indicated in Table 1. As is evident from Table 1, the different particle types cause different types of pathological effects despite their deposition in the same target tissue, namely the airspaces of the lung. Protracted exposure to the different particle types results in quite different types of pathological response.

**Table 1:** Particle-specific adverse health effects of the 3 particle types under consideration.

	**Adverse effects**	
**Particle**	**Pulmonary**	**Extra-pulmonary**
Asbestos	Interstitial fibrosis, bronchogenic carcinoma, pleural mesothelioma, pleural fibrosis, pleural plaques	Peritoneal mesothelioma, Autoimmune disease

Quartz	Nodular fibrosis, small airways disease, bronchogenic carcinoma, pleural fibrosis	Autoimmune disease

PM_10_	Increased lung cancer risk Exacerbations of COPD, Development of COPD Exacerbations of asthma	Deaths and hospitalisations for cardiovascular disease,

## Similar effects of particles *in vitro*

Numerous in vitro studies indicate that asbestos, quartz and PM_10 _have similar activities in cells *in vitro *– via oxidative stress, activation of NF-κB, pro-inflammatory effects and oxidative adduct formation (Table 2) [3-24]. We submit that this paradox, i.e. the mismatch between the similarities in effect seen *in vitro *and the differences in disease seen *in vivo*, poses a threat to the utility of in vitro nanotoxicology.

**Table 2:** Studies showing significant effects of the different particle types on inflammatory, genotoxic and oxidative stress endpoints in vitro

	**Endpoint**							
**Particle**	**Oxidative stress**	**NF-κB activation**	**AP-1 Activation**	**Chemokine production epithelial cells**	**TNFα production macs**	**Growth factor production**	**Direct genotox-icity**	**Apoptosis**

Asbestos	[21]	[4]	[23]	[16]	[8]	[14] fibroblasts	[15]	[3]

Quartz	[9]	[17]	[18]	[20]	[6]	[24] epithelial cells	[35]	[12]

PM_10_	[19]	[13]	[22]	[65]	[5]	[7] epithelial cells	[11]	[10]

In addition, there are other examples where *in vitro *testing has been shown to misclassify health hazard. The first would be glass fibres. Glass fibres have been reported to be positive in cell testing studies: generating reactive oxygen species, causing oxidant stress, causing DNA damage, inducing chromosomal aberrations, causing multinuclear formation, and inducing cell transformation [25]. However, due to lack of biopersistence, glass fibres have been reported to exhibit a low pathogenic potential in animal models. A similar false positive *in vitro *result has been reported for kaolin, being as cytotoxic as quartz in cell studies but exhibiting substantially lower fibrogenicity in exposed workers [26]. Lastly, a false negative has been reported for purified single-walled carbon nanotubes, which do not generate oxidant production or induce extensive toxicity in cultured macrophages but result in progressive interstitial fibrosis in mice exposed by pharyngeal aspiration or inhalation [27,28].

## Fibrosis

Exposure to all 3 particle types discussed in table 1 causes fibrosis, associated with accumulation of connective tissue cells and their products. At the cellular level, exposure to these particles is associated with oxidant production, activation of macrophages, sustained release of inflammatory mediators and growth factors, and activation of extracellular matrix production by fibroblasts. There are, however, differences in the nature and site of the fibrosis with the different particles. Asbestos causes predominantly an interstitial fibrosis in the lung parenchyma and small airways fibrosis [29]. Pleural plaques are also fibrotic lesions composed almost entirely of collagen, which occur on the parietal pleura in asbestos-exposed individuals [29]. In contrast, quartz is best-known for its association with nodular fibrosis (silicosis) of the lung parenchyma. Silica may also cause small airways fibrosis and pleural fibrosis but it is not characterized by pleural plaques. The association of PM_10 _with COPD in chronic exposure studies and a pathology study by Churg et al. [30] strongly implicate PM_10 _in causing airway fibrosis. It is not immediately clear why PM should cause airway fibrosis, yet not interstitial or nodular fibrosis, except perhaps that the severity of the inflammation caused by PM10 in a low exposure environmental situation is much less than that caused by quartz and asbestos in a high exposure occupational setting. Cigarette smoke does not cause nodular parenchyma fibrosis but does cause interstitial fibrosis [31]. Intrinsic differences in the shape, surface activity and composition of asbestos, quartz and PM_10 _might also explain differences in pathogenicity. Differences in site of deposition do not offer an explanation, as respiratory zone deposition can occur with quartz or asbestos and for the fine particles in PM and cigarette smoke [32]. The antioxidant defences in the parenchyma may be more effective against PM than quartz or asbestos, since quartz or asbestos act via direct membrane/particle interactions as well as oxidative stress. The high particle numbers in the ultrafine fraction of PM_10 _may also lead to more interaction with the pulmonary epithelium in the case of PM, which may result in more epithelial injury or interstitialisation in the case of the latter, both of which could culminate in fibrosis.

## Bronchogenic carcinoma

All 3 of the particle types addressed here have been associated with bronchogenic carcinoma (lung cancer). This is consistent with the airway epithelium as a primary site of deposition and therefore of highest dose. There could be differences in the mechanism of cancer, with the 3 particle types. Asbestos and quartz are essentially insoluble and so there are no readily identifiable lipophilic components that could enter the cells and form adducts with the epithelial cell DNA. Both asbestos [33] and quartz [34] can contain transition metals that could redox cycle and produce hydroxyl radicals which can form oxidative adducts like 8 hydroxy deoxyguanosine and activate signalling pathways which alter the balance between proliferation and apoptosis [35-37]. PM_10_, by contrast contains combustion-derived nanoparticles (soot) that contain PAHs along with other organics derived from fuel and its combustion. These have the potential to diffuse from the particle surface into cells and cause bulky DNA adducts.

## Cardiovascular effects

This is seen only with PM_10 _and seems a special case of effects distal to the lungs. The most likely explanation is that PM_10 _exposure, unlike asbestos and quartz exposures, which occur in a workplace, is the only particle exposure that occurs in susceptible people with cv disease. In patients with severe cardiovascular disease, even a mild systemic oxidative stress or inflammatory effects arising in the lung can impact the cv system [38] and precipitate an acute coronary syndrome. This is supported by studies demonstrating clear adverse effects of acute diesel particle exposure on the endothelium [39] and on ischaemia in the ventricular myocardium [40]. In addition, pulmonary exposure to residual oil fly ash, a component of PM, has been shown to augment adhesion of PMN to systemic microvessels, induce ROS generation in these vessels, and decrease the ability of these microvessels to respond to dilators [41]. Additionally, the combustion-derived NP particles contained in PM_10 _may be able to enter the circulation, especially in compromised individuals, and directly adversely affect the endothelium/plaques [42].

## Pleural and peritoneal effects

Pleural mesothelioma Mesothelioma is virtually unique to asbestos exposure, not being caused by any of the other particles. The target cell for mesothelioma is the mesothelium of the pleura or the peritoneum. It seems likely that asbestos fibres need to translocate to the pleural tissue to have this transforming effect and of course peripheral alveoli lie sub-pleurally and so any fibres that reach these peripheral alveoli and interstitialise, are close to the mesothelium. The pleura is 'reactive' to inflammation in the lungs [43] and the mesothelium undergoes proliferation when there is inflammation in the lung tissue [44]. Inflammation can be detected in the pleural space after lung exposure [45] to asbestos, and oxidative burst from the inflammatory leukocytes could be involved in producing mutation-forming oxidative adducts in the mesothelium but this has never been investigated.

Peritoneal mesothelioma This is an highly unusual tumour that, like its partner pleural mesothelioma, is only found with asbestos exposure. Since no other lung exposure, or lung disease, causes such a carcinogenic effect at a distal site, this argues for translocation of asbestos fibres to the peritoneal cavity. Other arguments, that oxidative stress or growth factor release from the lung reaching a sensitive site in the peritoneal mesothelium, are unlikely to be unique for asbestos alone.

Pleural fibrosis Both asbestos and quartz cause pleural fibrosis but PM_10 _does not. Quartz is not, however, considered to be especially tropic for the pleura and so this may be a result of reactive changes due to high levels of growth factors produced locally in the peripheral parenchyma. PM_10 _does not cause pleural fibrosis and this is consistent with the relative paucity of impact that PM shows for the parenchyma, given the close association between the peripheral alveoli and the pleura.

Pleural plaques Only asbestos causes pleural plaques, thin plaques of acellular collagen on the parietal pleural surface. These are likely a reflection of translocation and the lymphatic, or other, delivery of fibres to the pleural space and the failure of longer fibres to be cleared via the stomata in the parietal pleura, the normal site for egress of lymph from the pleural space. Accumulation at these exit points of fibres on the pleura is likely to upset the normal serosal fibrinogenesis balance [46] allowing for a fibrinous exudates to form a scaffold for fibrosis. Alternatively, the presence of fibres on the mesothelium at the stomatal openings could set up a localised inflammatory response that is further 'irritated' by breath movements and the friction of the movement of the pleural surfaces over one another with maximal contact at the raised areas of the ribs. These events may combine to produce the characteristic highly localised fibrotic lesions of pleural plaque overlying the ribs.

## COPD

COPD is a complex syndrome of two important pulmonary diseases-bronchitis and emphysema – that combine to produce airway narrowing. Although classically it is caused by cigarette smoking, the new definition includes gases and dusts as causative agents [47]. Airflow limitation is the characteristic manifestation of COPD, documented as a decline in FEV_1_. However there is another hallmark of the disease, namely, exacerbation. This is defined as a sudden worsening of the condition, often necessitating hospitalisation. PM_10 _has been implicated in causing one aspect of COPD – small airways fibrosis [30] and in causing exacerbations of COPD [48]. In one study, autopsy lungs from age-matched non-smoking females with no history of working in dusty occupations from a high PM_10 _pollution area Mexico City were compared with lungs from a similar population from low PM_10 _area – Vancouver [30]. Using morphometric techniques, considerable fibrosis was detected in the small airways of the Mexico City dwellers but very little of this type of pathology was seen in the Vancouver dwellers. This is evidence that PM_10 _causes one aspect of COPD, namely the fibrotic thickening of the small airways that contributes to airways obstruction. There are many time-series studies demonstrating that increases in PM10 cause exacerbations of COPD in the following hours [49]. There is no evidence that asbestos or quartz cause development of classical COPD (emphysema plus bronchitis with exacerbations) nor that either of these mineral particles causes exacerbation in existing COPD patients.

## Asthma

Neither quartz, asbestos nor PM_10 _appear to 'cause' asthma in that there is no greater incidence of asthma amongst either workers or the general populations exposed to these particles. Increases in PM_10 _are, however, associated with increases in exacerbations of asthma in the hours following increased ambient PM exposure. The 'healthy worker effect' may explain this, in that no individual with airflow limitation would work in a dusty trade as they may anticipate or actually experience additional respiratory compromise from the dust exposure. In either case, they are unlikely to remain employed in such an industry and will seek alternative employment. This is not, of course, the case for PM_10_, where everyone, including asthmatics, experiences exposure with no choice in the matter.

## Extra-pulmonary effects

Autoimmune disease Autoimmune disease is a common sequel of chronic inflammatory diseases [50,51]. It is seen in quartz and asbestos-exposed individuals [52] and may be a result of the severity of the inflammation seen with occupational exposure to quartz and asbestos. Lack of autoimmunity with PM_10 _exposure may reflect the relatively mild inflammation seen with PM_10_, supported by studies with concentrated ambient particles (CAPs) where only mild inflammation is seen even with CAPs at tens of times the ambient level [53].

Cardiovascular deaths and hospitalisations Exposure to PM_10 _in both chronic and short-term studies is associated with deaths and hospitalisations for cardiovascular causes [40,54]. The impact of particle inhalation on atherothrombosis, the principal cause of cardiovascular morbidity and mortality, is not well understood but there are a number of hypotheses. Pulmonary inflammation may be able to influence events in the atherosclerotic lesions in the vascular wall, which are also inflammation-driven [55]. Certainly acute effects such as endothelial dysfunction and ventricular ischaemia are pronounced in the short-term after exposure to diluted diesel exhaust in chamber studies [39,56]. Animal studies suggest that inflammation caused by pulmonary deposition of diesel particles can enhance the thrombogenicity of the blood [57]. Nurkiewicz et al. [41] have also reported that pulmonary exposure to residual oil fly ash causes PMN adherence and ROS production at systemic microvessels. In addition, nanoparticles in PM_10 _may be able to enter the circulation and directly affect the atherosclerotic plaque or the overlying endothelium [58].

## Similar effects of the different particles in vitro

It is clear from the above that the 3 different particle types cause a spectrum of adverse effects both in the lungs and at extra-pulmonary sites. This contrasts with the distinct similarities in the nature of the responses seen in *in vitro *studies with the same 3 particle types. This is shown in Table 2. All 3 particle types, when added to cells in culture, show abilities to cause oxidative stress, activation of redox-sensitive transcription factors, cytokine and chemokine production, growth factor release, and direct genotoxicity.

Oxidative stress is a strong over-arching theme in the cellular effects of particles. In general, the responses to particles can be understood as responses to oxidative stress. Most pathogenic particles appear to cause oxidative stress in target cells. The ubiquitousness of this effect is striking and may reflect some fundamental response of cells to foreign surfaces inside the cell as well as the obvious redox-cycling of some particle components like organics [59] and transition metals [60]. Oxidative stress is a signalling system in cells that leads to gene expression [61]. Increasing levels of oxidative stress inside cells caused by particles may result in graded response from anti-oxidant defence induction via NF-κB and AP-1, through pro-inflammatory signalling via the same transcription factors to induction of apoptosis [1]. Therefore it is something of a self-fulfilling prophecy that adding particle to cells will cause oxidative stress if the dose is high enough, and that if there is oxidative stress there will be NF-κB and AP-1 activation. The activation of the transcription factors is tightly related to redox balance in the cell. There may be exceptions, however, and in our hands quartz and raw carbon nanotubes (metal contaminated) cause the same degree of glutathione depletion but only quartz causes NF-κB activation. Therefore particle-derived oxidative stress alone may not be sufficient but may often be accompanied by one or more other signals to the cells from the particles for transcription. It has been proposed that the more pathogenic the particle, the more it causes oxidative stress [1]. However, this relationship does not hold for purified (low metal) single-walled carbon nanotubes. Although raw single-walled carbon nanotubes have been shown to be toxic to bronchial epithelial cells in vitro, ROS generation and oxidant damage appear dependent on the presence of contaminating iron [62]. Indeed, in vitro exposure of macrophages to purified single-walled carbon nanotubes failed to induce ROS production or stimulate production of inflammatory cytokines [27].

Release of chemokines, cytokines and growth factors can be grouped together as they are all secretions from cells, normally leukocytes, that induce inflammation or mesenchymal cell activation or proliferation. Once again all of the particles considered here when added to one target cell or another stimulate the gene expression and release of these mediators. Macrophage-derived cytokines, such as TNFα, act on epithelial cells to enhance their chemokine response to particles [63,64]. Many cytokines and chemokines, such as IL-8 [65,66] and TNFα [67] have NF-κB motifs in their promoter and are oxidative stress-responsive and so their activation is to be anticipated, if oxidative stress is present.

Genotoxicity and apoptosis are linked together because of the relationship between genotoxicity and the induction of apoptosis [68]; again there is a common role for oxidative stress in both processes. However, purified single-walled carbon nanotubes appear to be an exception to this rule, since inhalation exposure in mice caused k-ras gene mutation without persistent oxidant stress [28].

## Conclusion and relevance for predictive toxicology of nanoparticles

**Figure 1:**
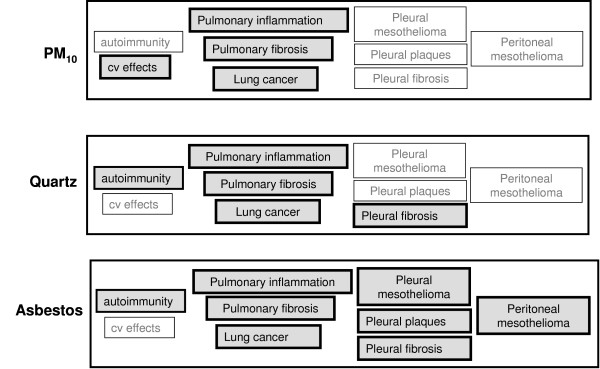
**How different particle types affect different pathological compartments; where there is an impact the typeface is bold and the box outline is thicker**.

Taken together, these in vitro data tell us that oxidative stress is a central tenet of the current hypothesis on the action of particles at the cellular level. This is in keeping with understood actions of a large number of chemicals, such as metals, organics, etc., that also act by causing oxidative stress in various target tissues and organs [69-75] (Figure 1). This suggests that the information that is garnered from a study that demonstrates that a particle can cause oxidative stress *in vitro *may be of highly questionable value. The real question is whether different particles have different abilities to deliver oxidative stress and possibly the nature of the oxidative stress or its site of delivery both inside the cell and in target tissues distant to the airspace surface on which the particle deposits. The latter implies a need for toxicokinetics to understand plausible dose in any target tissue. Such toxicokinetics as has been carried out on particles suggests, for example, that particle size is related to translocation, and that larger particles do not on the whole translocate to any appreciable degree, except for asbestos. This seems evident from Figure 1, where asbestos seems to be able to translocate most efficiently and has the smallest or at least thinnest particles.

At the moment the particle toxicology world is dominated by the issue of nanotoxicology and the effects of nanoparticles. The experience with combustion-derived nanoparticles and PM_10 _and some studies with manufactured nanoparticles suggest that there could be risk from the development and use of manufactured nanoparticles. There are many new particle types that await testing, but there is no great appetite for animal testing. Thus, there is a perceived need for *in vitro *testing that can predict the hazard. It might be tempting to carry out *in vitro *toxicology alone, and it might not be surprising to find that some of these particles cause oxidative stress and NF-κB activation etc. [1]. However, purified single-walled carbon nanotubes appear to be an exception to this general rule [27]. In this case, direct action on lung fibroblasts (increase proliferation, augmentation of collagen production, and induction of cytokine message) rather than oxidant stress may explain the in vivo response (interstitial fibrosis) to single-walled carbon nanotubes [76,77]. The content of the present paper, however, suggests that great care should be taken in interpreting in vitro data. A particle that causes oxidative stress to cells in culture could, in theory, behave like quartz, asbestos or PM_10_, and it would not be evident which of these it would behave like, from the *in vitro *data.

Short of waiting for the diseases themselves to develop, what can we do to assist in bridging the gap between in vitro and disease prediction, Dosimetry data is one way forward but this is notoriously difficult and there is no mass balance toxicokinetic data available for any particle. It seems unlikely that PBPK modelling can adequately predict nanoparticle dosimetry, although there are ongoing efforts. Toxicokinetics is difficult and expensive but offers the only way forward in order to put *in vitro *studies on a relevant and plausible footing with regard to dose to use, target cells to study, and ultimately, the usefulness in hazard identification.

None of the above is meant to say that in vitro toxicology isn't valuable for understanding mechanisms. It is absolutely vital for this purpose and has formed the backbone of the authors' research. However, in vitro research cannot replace some aspects of animal testing, and there is need for investment in good nanotoxicology research across all aspects of the discipline to gain the advances in understanding that are needed.

In addition to providing toxicokinetics and dosimetric data, *in vivo *testing of nanoparticles also remains important because of the possibility of picking up a novel pathology or target tissue. In vivo testing also sidesteps the difficulty in linking "generic" cellular responses to different pathologies but is also needed because no, or only poor, in vitro alternatives currently exist for many "systemic" responses such as cardiovascular responses, immune responses and syndromes such as COPD. We need to continue to invest in in vivo testing because many diseases linked to nanoparticles are systemic in nature. In addition the distribution of nanoparticles in different organs and tissue is poorly studied. At the same time, toxicologists need both in vivo and in vitro models to study the pathogenesis of these diseases and the behaviour of nanoparticles at all levels, organismal to sub-cellular. Only limited progress has been made in developing in vitro models to study the distribution and translocation of nanoparticles across the different barriers. More effort is urgently needed develop more specific in vitro models that will enable us to understand systemic effects of nanomaterials.

## Competing interests

The authors declare that they have no competing interests.

## Authors' contributions

All of the authors contributed, in equal measure, their experience in the toxicology of fibres and particles in the conception and writing of this review. All authors read and approved the final manuscript.

## Disclaimer

The findings and conclusions in this report are those of the authors and do not necessarily represent the views of the National Institute for Occupational Safety and Health.
